# Assessment of the relationship between pre-chip and post-chip quality measures for Affymetrix GeneChip expression data

**DOI:** 10.1186/1471-2105-7-211

**Published:** 2006-04-19

**Authors:** Lesley Jones, Darlene R Goldstein, Gareth Hughes, Andrew D Strand, Francois Collin, Stephen B Dunnett, Charles Kooperberg, Aaron Aragaki, James M Olson, Sarah J Augood, Richard LM Faull, Ruth Luthi-Carter, Valentina Moskvina, Angela K Hodges

**Affiliations:** 1 Depts. of Psychological Medicine and Medical Genetics, School of Medicine, Cardiff University, Cardiff, UK; 2 Ecole Polytechnique Fédérale de Lausanne (EPFL), Lausanne 1015, Switzerland; 3 Fred Hutchinson Cancer Research Center, Seattle, WA 98109, USA; 4 Dept of Statistics, University of California, Berkeley, CA 94720-3860, USA; 5 School of Biosciences, Cardiff University, Cardiff, UK; 6 MassGeneral Institute for Neurodegenerative Disease (MIND), Massachusetts General Hospital, Charlestown MA 02129-4404, USA; 7 Dept of Anatomy with Radiology, University of Auckland, uckland City Hospital, Auckland, New Zealand

## Abstract

**Background:**

Gene expression microarray experiments are expensive to conduct and guidelines for acceptable quality control at intermediate steps before and after the samples are hybridised to chips are vague. We conducted an experiment hybridising RNA from human brain to 117 U133A Affymetrix GeneChips and used these data to explore the relationship between 4 pre-chip variables and 22 post-chip outcomes and quality control measures.

**Results:**

We found that the pre-chip variables were significantly correlated with each other but that this correlation was strongest between measures of RNA quality and cRNA yield. Post-mortem interval was negatively correlated with these variables. Four principal components, reflecting array outliers, array adjustment, hybridisation noise and RNA integrity, explain about 75% of the total post-chip measure variability. Two significant canonical correlations existed between the pre-chip and post-chip variables, derived from MAS 5.0, dChip and the Bioconductor packages affy and affyPLM. The strongest (CANCOR 0.838, p < 0.0001) correlated RNA integrity and yield with post chip quality control (QC) measures indexing 3'/5' RNA ratios, bias or scaling of the chip and scaling of the variability of the signal across the chip. Post-mortem interval was relatively unimportant. We also found that the RNA integrity number (RIN) could be moderately well predicted by post-chip measures B_ACTIN35, GAPDH35 and SF.

**Conclusion:**

We have found that the post-chip variables having the strongest association with quantities measurable before hybridisation are those reflecting RNA integrity. Other aspects of quality, such as noise measures (reflecting the execution of the assay) or measures reflecting data quality (outlier status and array adjustment variables) are not well predicted by the variables we were able to determine ahead of time. There could be other variables measurable pre-hybridisation which may be better associated with expression data quality measures. Uncovering such connections could create savings on costly microarray experiments by eliminating poor samples before hybridisation.

## Background

Conducting microarray experiments using Affymetrix arrays is expensive. The quality of the starting material, for instance human post-mortem tissues, is often predetermined and samples may be scarce, leading to variable quality of the extracted RNA. We set out to explore the relationship between quality control (QC) variables used to assess samples prior to hybridisation (pre-chip) and those used to assess the quality of the hybridisation and resulting microarray data (post-chip). We sought better to define which variables were important in determining the quality of the final data and to see in turn whether any post-chip measures could predict pre-chip variables.

Examination of quality in GeneChip experiments has been hampered by the relatively new technology, rapidly changing platforms (chip types) and the inability of most centres, because of expense, to run large series of samples to examine the characteristics and limitations of the technology. In addition, the output of the QC measures reflects both technical variation in the performance of the experiment and the biological variation of the samples available. Affymetrix give a series of guidelines about threshold values for quality control measures produced in the RPT file by their algorithm (GCOS or MAS 5.0) [1]. They do not really indicate what the user should do if the quality control measures fall outside their recommendations except for omitting the sample from the analysis.

Dumur et al. [2] examined technical variables, the effect of freezing cRNA and that of running the same samples on different days and found that freezing had little effect but hybridisation date did affect GeneChip QC variables. They examined 18S/28S ratios in total RNA but found that these did not appear to predict the efficiency of the cRNA synthesis. Finkelstein [3] reported on improvements over time in the quality of expression data obtained in the St Jude's Children's Research Hospital from 2000–2004, with over 5000 GeneChips hybridised. Finkelstein attributes the improvements to technical advances in hardware and software from Affymetrix and the increasing experience of the centre: technical variations clearly had the most impact on chip data in this longitudinal retrospective analysis. No attempt was made in this study to examine the pre-chip variables that influence the GeneChip performance. However, in studies examining the effect of biological variability in the samples, the RNA integrity of samples from human brain was found to influence gene expression profiles profoundly; in turn RNA quality was influenced by agonal state but not gender, age or post-mortem interval (PMI: number of hours from death until tissue preservation) [4,5].

We examined gene expression in a large series of RNA samples extracted from post-mortem human brain [6]: technical variation was minimised as far as possible. All GeneChips came from two manufacturing batches, one person carried out all the reactions and the same instrumentation was used for all hybridisations. There are only limited variables that can be examined before GeneChips are hybridised. We assessed total RNA quality subjectively, using Agilent Bioanalyser traces, as well as with the Agilent-derived RNA integrity number (RIN) [7]. We excluded some samples based on their subjectively assessed RNA quality as RIN became available only after the samples had been hybridised (we later generated RIN from the Agilent traces). We also examined the effect of cRNA yield and PMI. We derived post-chip QC measures from MAS 5.0 [8], dChip [9] and the Robust Multichip Average (RMA) algorithms [10,11]. We used these measures as a quality control filter, initially in a subjective way, to exclude samples that had been hybridised to GeneChips from further analysis if they were regarded as outliers in the algorithms above [6].

We have now used these data to examine the relationship of pre-chip variables to post-chip quality control measures. Although we believe our original subjective decision to exclude samples at each step in the process from sample collection to expression analysis was justified [5], these decisions were based on limited understanding of the relationship of the quality control measures to each other and to the quality of expression data. By exploring the relationship between the various pre- and post-chip quality control measures, we hope to move towards a more objective assessment, combining key quality control measures. This would reduce the chances of erroneous sample exclusion or inclusion with its concomitant effect in the reduction of power to detect altered mRNA levels between the experimental conditions.

## Results

### Effect of brain region

The samples we used were derived from a series of HD and control brains from the New Zealand Neurological Foundation Human Brain Bank. The full consent of all families was obtained at the time of autopsy and the University of Auckland Human Subjects Ethics Committee approved the protocols used in these studies. For most brains RNA was isolated from three regions: caudate nucleus (CN), cerebellum (CB) and motor cortex (MC). Because there was a significant effect of brain region on some of the pre-chip (RIN and cRNA yield) and post-chip variables (SF and RawQ) (see methods for a detailed explanation of the pre- and post-chip variables) we adjusted all variables for brain region (see methods). All further analyses were carried out with the adjusted variables.

### Pre-chip variables

**Figure 1:**
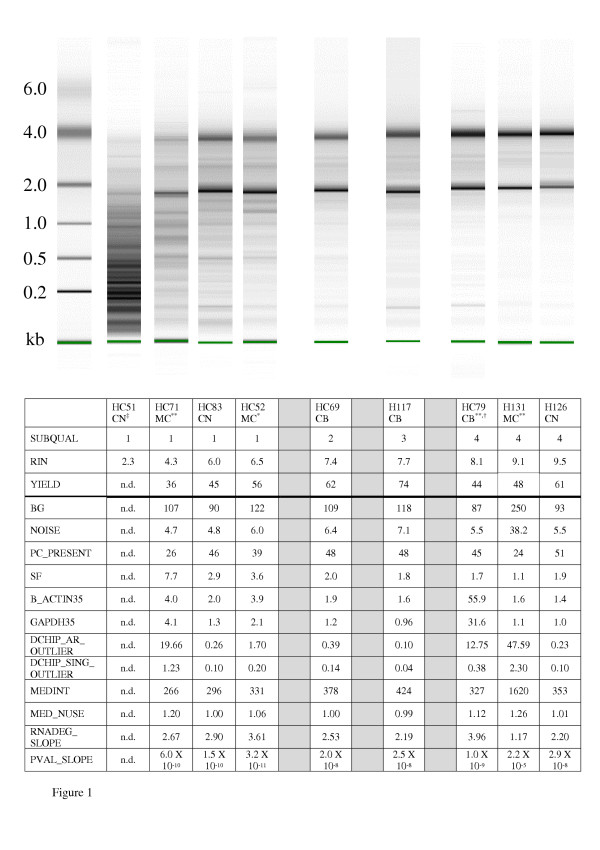
**Total RNA Gel images from the Bioanalyser (Agilent)**. Representative total RNA samples of varying quality, as assessed objectively by RIN and by subjective assessment. Selected corresponding pre- and post-chip variable assessments are also shown. Samples identified as outliers on post-chip quality control measures but not excluded are labelled (*); outliers excluded from expression analysis are labelled (**). Sample from case with prolonged agonal state (†). Sample not run on arrays due to poor quality total RNA (‡). n.d. is value not determined. CB, cerebellum; CN, caudate nucleus; MC, motor cortex.

The pre-chip variables included two assessments of RNA quality: a four-category subjective visual assessment of Bioanalyzer traces (SUBQUAL) carried out by two of us on all samples (AH, RL-C) and the Agilent-derived RIN, which only became available after the GeneChips had been hybridised and thus was generated retrospectively (Figure 1). Subjective quality assessment was based on ribosomal peak definition and apparent presence of low molecular weight RNA (see methods). Figure 1 shows that samples HC79CB, H131MC and H126CN, graded as excellent (SUBQUAL = 4), have well resolved ribosomal RNA peaks and virtually undetectable low molecular weight RNA. HC117CB was graded good (SUBQUAL = 3) and shows well-resolved rRNA bands but has a faint trace of low molecular weight RNA. HC69CB was graded moderate (SUBQUAL = 2); it has a less distinct 28S rRNA band than seen at higher grades with visible low molecular weight RNA. The remaining four samples were assessed as poor quality RNA (SUBQUAL = 1). Here the 28S rRNA band is blurred or invisible and there is substantial evidence for the presence of low molecular weight RNAs. We also investigated the effects of PMI and cRNA yield in the final step of target preparation. Acceptable ranges and details of the values obtained for our samples are given in Table 1 and illustrated in Additional File 1.

As expected from post-mortem brain tissue, where there is little control over the events leading up to availability and preservation of tissue, PMI and RNA integrity as measured by subjective and objective assessment were variable (Table 1). In the original study a small number of samples (N = 9) were excluded on the basis of subjective total RNA quality, determined as above, and were therefore not hybridised to arrays. Although they were samples at the low end of the RNA quality spectrum, this decision was somewhat arbitrary and driven by array availability rather than their clear outlier status. A few samples of approximately equal subjective quality to those not hybridised to arrays performed well on all post-chip quality control assessments (Figure 1, sample HC83 CN). Although sample reactions were normalised with a reaction input of 10 μg, subsequent cDNA and cRNA reactions resulted in variable yields of cRNA. A few samples (N = 4) were excluded because they did not meet the minimum cRNA yield of 15 μg recommended by Affymetrix. Three of these samples were originally rated subjectively as having poor quality RNA.

PMI correlated moderately negatively with our subjective assessment of RNA quality (treated as a numerical variable), with the Agilent RIN and with cRNA yield (Table 2). As expected, subjective RNA quality was highly correlated with the Agilent RIN (correlation coefficient 0.71, p = 0.000001; ANOVA F for linear trend 110.0 on 1 and 108 df, p = 3.6 × 10^-18^). Subjective quality and RIN also correlated significantly with yield. Figure 1 shows the subjective assessment of RNA quality for a representative selection of RNA samples of the entire quality spectrum along with the corresponding RIN. The four subjective categories had considerable overlapping values for RIN: the best RNA category had a RIN range of 7–9.5 and the poorest RNA category had a range of 4.3–7.7, with the other two categories between these two.

### Post-chip variables

We computed quality measures using three different softwares: MAS 5.0 [8], dChip [9] and the Bioconductor packages affy and affyPLM [10,12,13] (see methods and Table 1). For most of these measures the relationship between the measure and quality of quantitative expression data is unknown. There is guidance about acceptable limits for some measures, but currently the decision to exclude data from analysis is empirical and generally based on outlier status within each experiment: that is, chips that do not look like other chips in the analysis are excluded. In our experiment ten chips (out of 117) were flagged as outliers as they were found to be outliers on multiple post-chip variables (Table 3 and see Additional File 2). This was a subjective decision based on recommended acceptable ranges for each QC measure in the algorithms and the extent to which the value for a particular sample fell outside the interquartile range for that measure in the experiment (see Table 1). For some of the measures, e.g. RawQ, although most of our samples were outside the recommended range, there was little variation across the experiment. All ten of the chips flagged as outliers were outside the acceptable range recommended for MED_NUSE and those eventually excluded had MED_NUSE ≥ 1.08 (Table 3). Measures that identified the same samples as outliers were: PC_PRESENT, DCHIP_SING_OUTLIER, MED_NUSE, NOISE, BG and MEDINT suggesting that to some degree they may be assessing similar characteristics. Six of the identified outlier samples judged to have a combination of the most extreme values were subsequently excluded from analysis. The other four samples flagged as outliers were not excluded from analysis because they had less severe DCHIP_AR_OUTLIER and MED_NUSE values and were not outliers on more than three other measures (Table 3 and see Additional File 2).

**Figure 2:**
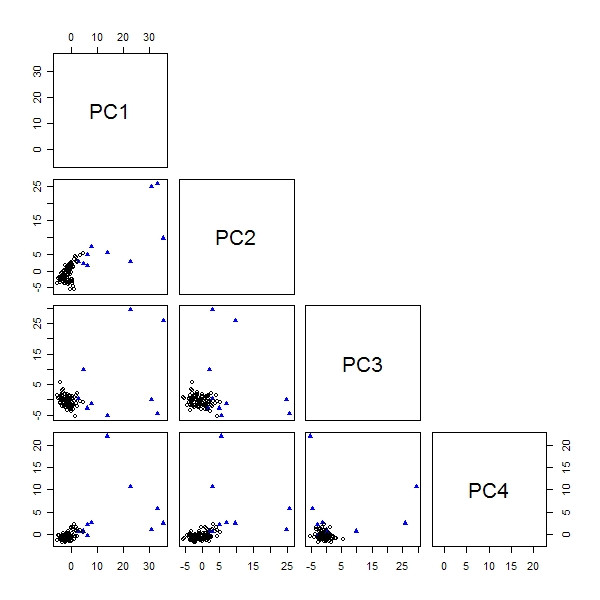
Pairwise scatterplots for the first four principal components. Outlier chips (Table 3) are represented by blue triangles.

The different algorithms generating the post-chip variables measure aspects of the same underlying hybridization process. To explore the relationships between the variables generated by the different algorithms we carried out a principal component analysis using data from U133A chips hybridised in the experiment for which measures of all variables were available (N = 112). Since many of the pre- and post-chip variables were not normally distributed, we based the principal component analysis on Spearman (non-parametric) correlations. The first 4 components explained 75% of the variation (Table 4 and Figure 2) with the remaining components each assessing < 6%. The principal component analysis (Table 5) demonstrates that the different QC algorithms assess a series of very similar parameters from the chips. The RMA-QC and dChip algorithms assess the variation between chips by comparing all chips: these measures form the first principal component and account for around 1/3 of the total variance. In contrast, MAS 5.0 computes single chip measures. The MAS 5.0 scaling factor (SF) and the RMA-QC median log ratio measure of bias (B_LR) contribute to the second component: these measures reflect (in different ways) how much adjustment of the total signal is required to make one array comparable with the rest. The second component also indexes the negative correlation between high bias and low percentage present calls. The percentage present call in both MAS 5.0 and dChip appear to behave similarly, with high scaling factor or bias values going together with low percentage present calls. MAS 5.0 and dChip assess 'noise' (third component), seemingly in a very similar way, accounting for around 13% of the variance. MAS 5.0 quantifies RNA integrity using 3'/5' ratios of two index genes (B_ACTIN35, GAPDH35); the Bioconductor affy package attempts this more globally by examining the 3'/5' ratio along the probes for all probesets (RNADEG-SL, PVAL_SLOPE): this fourth component accounts for 12% of the variance in post-chip measures. These four principal components account for 75% of the total variance and thus index the measures that are most important among those considered in examining the array expression summaries.

**Figure 3:**
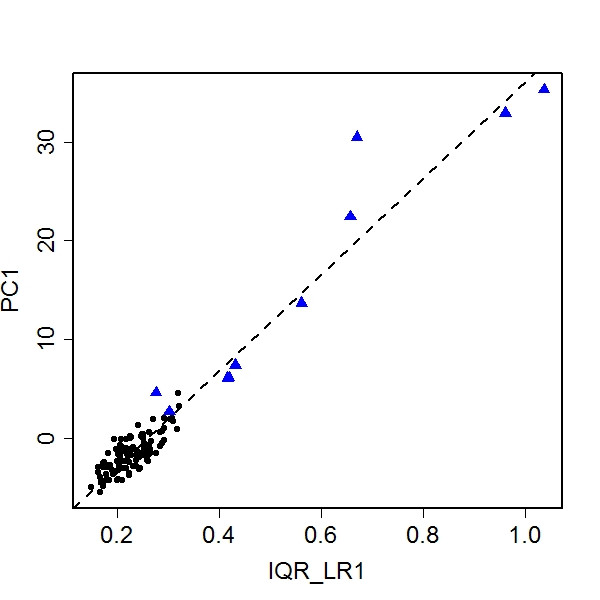
Pairwise scatterplots of PC1 vs IQR_LR1. Outlier chips (Table 3) are represented by blue triangles.

The first principal component (PC1) is highly correlated with variables which include IQR of relative log expression (IQR_LR 1 and 2, and IQRplusAbsB_LR 1 and 2). This quantity measures the variability in the degree to which a given chip differs from a virtual median chip – that is, a chip which would have median expression for each probe set. Consistent with the property of principal components of capturing the variability in the data, those chips with highest IQR relative log expression tend to be those which are largest on PC1 (Figure 3).

The largest outliers in Figure 3 are H131MC, HC71MC, H123CN, H122CB, HC79CB, HC52MC, HC55MC and H85CB. Examination of quality measures for these samples (Table 3) indicates that the chips are outliers for a few different reasons rather than for the same underlying cause. Some of them would appear to have undergone some degradation, particularly those with low SUBQUAL and RIN (e.g. H122CB, HC52MC, HC55MC), while others probably failed at some point of the hybridisation (e.g. H131MC, H123CN).

### Relationship of pre-chip to post-chip variables

Canonical correlation analysis was then performed in order to detect relationships between the two sets of variables: the set of 4 pre-chip and that of 22 post-chip variables. Four canonical correlations were calculated (Table 6). These were used to investigate whether any of the pre-chip variables were associated with the post-chip variables in a way that would allow decisions to be made about whether samples are of suitable quality to hybridise to an array and give meaningful results.

The first canonical correlation (0.84, p < 0.001) indexes nearly all of the relevant relationships between the pre- and post-chip variables: it is highly significant (Table 6). The second canonical correlation (0.59, p = 0.012) is also significant but indexes much less of the relationship between pre and post-chip variables. Post-mortem interval makes very little contribution to either the first or second canonical correlations (Table 7). RIN (or subjective quality) and yield are, however, both important (Table 7). Of the RMA QC measures, the MED_NUSE and B_LR, IQR_LR and IQRplusAbsB_LR are all negatively correlated with yield and RIN (Table 8). In MAS 5.0, SF and the 3'/5' ratios are negatively correlated with yield and RIN, and % present is positively correlated. So the lower the yield or RIN, the higher the SF, median NUSE or bias and the 3'/5' ratios: that is, the more 'correction' needs to be applied to bring the errant chip in line with the others. Percentage present call is positively correlated with yield and RIN in both MAS 5.0 and dChip; this is likely to reflect the ability to detect signal when both amount and quality of sample are high. Most of the other post-chip variables are less highly correlated in the first canonical correlation (Table 8). In the second canonical correlation the relationships are considerably weaker and less significant. There is virtually no effect of RIN, though SUBQUAL is negatively correlated and YIELD positively correlated with measures of 3'/5' ratios and noise.

### Prediction of RIN using post-chip measures

Although estimation of RIN based on post-hybridization measures is generally not of interest for a lab analyzing its own data, it is potentially useful in the context of secondary analyses done by other labs. An increasingly common example is analysis by different groups of publicly available data, for example data deposited in Gene Expression Omnibus (GEO) [14,15]. In this case there may not be ready access to any pre-chip measures such as RIN. However, if the cel files are available, in the case of data derived from Affymetrix chips for example, then post-hybridization quality measures are readily computable. If the post-chip measures that best predict RIN can be identified, then confidence in excluding chips from secondary analyses on the basis of poor quality RNA could be made and thus improve the quality of data produced in any analysis. However, RIN alone was not able to predict outliers in the current study where samples had a range of RNA quality, and we could not determine an accurate threshold of RIN below which samples fail post-chip quality control.

**Figure 4:**
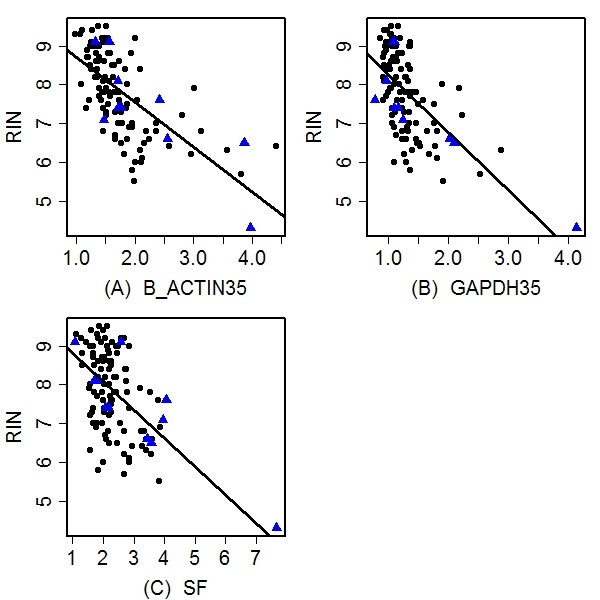
Pairwise scatterplots showing RIN plotted against (A) B_ACTIN, (B) GAPDH and (C) SF. Outlier chips (Table 3) are represented by blue triangles.

In our data, excluding the gross outlier HC79CB, the single variables most highly correlated with RIN are B_ACTIN35, GAPDH35 and SF. B_ACTIN35 and GAPDH35 are very highly correlated with each other (ρ = 0.99), with somewhat lower correlation with SF (ρ = .55 – .65). Robust regression [16,17] of RIN on B_ACTIN35 yields the predictor

estimated RIN = 9.8 - 1.1 * B_ACTIN35,

with a residual standard error of about 0.9. Ordinary regression yields nearly identical coefficients, both of which are highly significant (p < 6 × 10^-15^), and an adjusted R^2 ^of 0.42. Using GAPDH35 as the predictor yields

estimated RIN = 9.7 - 1.5 * GAPDH35,

with residual standard error of about 1.1. Again, coefficients estimated by ordinary regression are very close, with highly significant coefficients (p < 2 × 10^-12^) and adjusted R^2 ^of 0.36. Including SF as an additional term does not greatly improve the fit of the model. Using SF alone, we obtain

estimated RIN = 9.4 - 0.7 * SF,

with residual standard error of about 1.0, highly significant coefficients (p < 2.5 × 10^-9^) and adjusted R^2 ^= 0.27. SF may be easier to obtain than B_ACTIN35 or GAPDH35 for users without MAS/GCOS. Figure 4 shows the scatter plots with regression lines for each of these scenarios. The outlier chips (from Table 3 and Additional File 2) are indicated in the plot (with the exception of HC79B, which is a gross outlier on these measures). We thus see that RIN is at least moderately predictable from measures readily obtained from the MAS/GCOS rpt file and which are computable from the cel file.

### The effect of including chips that fail post-chip QC on analysis of differential gene expression

**Figure 5:**
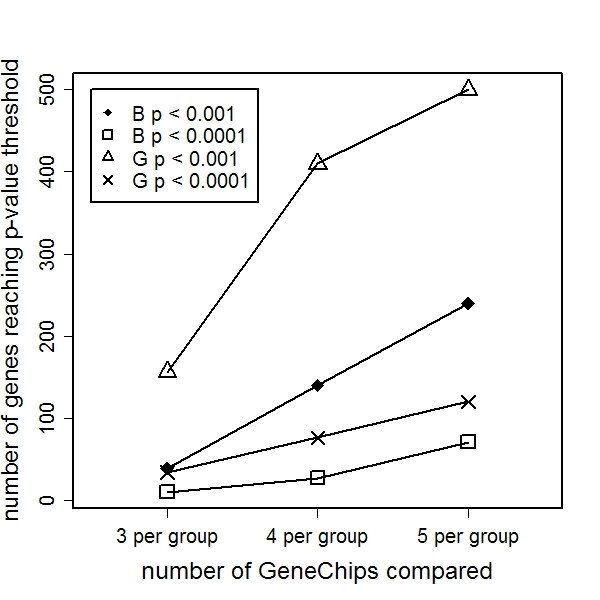
**The effect of including poor quality chips in analyses on ability to detect differential gene expression**. Fewer differentially expressed genes are detected when comparing male and female motor cortex if a chip that failed QC is included in the analysis, reflected in the at least 50% fewer probe sets detected as differentially expressed at the two different p-value thresholds (t-test nominal unadjusted p-values). Bad (B) indicates comparisons where one chip that failed QC (HC71: female) was included in the analysis; Good (G) indicates comparisons where all chips passed QC. Samples were matched for age. This effect is most marked with very small chip numbers and gradually becomes less as chip numbers increase.

Figure 5 illustrates the effect of including a chip that did not pass post-chip QC in the analysis on the detection of differential gene expression between males and females in cortex using different sample sizes (3, 4 or 5 per group). Here, either one chip that failed post-chip QC is included (the 'bad' group) or an age-matched 'good' chip is used so that we can assess the effect of quality on numbers of probesets detected as differentially expressed. Not surprisingly, the effect is most marked when only small numbers of replicates are available. The number of differentially expressed genes at the selected p-values is less than half the number detected when all the chips passed post-chip QC. As larger sample sizes provide greater power, the increased numbers of probesets detected as differentially expressed should be mostly due to a reduced number of false negative results.

## Discussion

It would be very useful to be able to predict which samples will go on to give interpretable results after hybridisation, based on their pre-chip quality control variables. In some cases, for instance when using post-mortem human tissue, over which the researcher often has little control before preparing the RNA, these considerations become paramount. We examined the results we had obtained using 117 brain RNA samples hybridised to U133A GeneChips to see if we could establish any relationships between pre- and post-chip variables. As it might also be useful for the interpretation of data from the publicly available resources we also examined whether we could predict the RIN from the post-chip measures.

The two measures of RNA integrity we used were highly correlated; these in turn correlated with cRNA yield indicating that more intact total RNA will lead to better yields at the end of the sample preparation process. Indeed, the few samples that did not reach the Affymetrix recommended 15μg cRNA yield were largely generated from poor quality RNA. As we did not hybridise those samples we judged to have RNA that was too degraded (by subjective assessment) or that did not generate sufficient cRNA we cannot judge their impact on our post-chip measures. There are no reports assessing the performance of the Agilent-generated RIN on GeneChip data quality to date. Using the post-chip quality control measures in our experiment, we found that samples with a RIN > 5.5 produced expression data of sufficient quality to be included in analyses. We found that longer post-mortem intervals were associated with poorer quality RNA (lower RIN or SUBQUAL) as might be expected, although the level of correlation is low, around -0.2. Tomita et al. [5] found that RNA integrity measures correlated well with their measure of post-chip performance but that PMI was not correlated with RNA integrity measures. In common with previous reports, they did find that agonal factors (e.g. terminal coma, pyrexia and coma) correlated better with RNA integrity than with PMI [4,18]. Of the three samples in our study derived from cases with prolonged agonal state, none were clear outliers with respect to RNA quality or cRNA yield compared with the rest of the RNA samples in this experiment. They are likely to be too few and the quality too variable in our experiment to separate them out at this point but all three were clearly outliers on post-chip measures. RNA integrity is also related to tissue pH [4,18-20] and pH has been used as a surrogate measure of RNA integrity as a result of pre- and post-agonal events. We did not measure this in our samples, but given the relative ease of doing this test, it would be a worthwhile measure in the absence of clinical information. The variation in RNA quality observed in our samples may in part be due to PMI and agonal state, but these factors seem to play only a small part in the variation of quality observed. Other factors such as technical problems (e.g. freezing and storage) and unknown physiological processes likely had a greater impact.

The post-chip measures can be generated from data available in the public databases, but the pre-chip quality control measures are usually not provided. Therefore it might prove useful to predict the RIN, as an objective measure of RNA integrity, retrospectively. It is not clear how generalisable the models generated from our data will be although they indicate that there may be relationships that can provide an estimate of this information. Only examination of a large number of varied data sets will give a true indication of their general validity. However, one of these predictors might be used to obtain a 'quick and dirty' estimate of the quality of RNA from which the data is derived. More importantly, it highlights the most appropriate post-chip measures that predict RNA quality (B_ACTIN35 and GAPDH35 and to a lesser extent SF) and gives an estimate of their relationship which can be used to select chips to exclude from analysis based on their outlier status in these variables which would improve data quality, particularly in small datasets or datasets combined from several experiments.

Yield of cRNA is significantly correlated with measures of RNA integrity. It is thus difficult to know if yield per se is related to PMI or whether this is simply a result of its relationship with RNA integrity. Although yield clearly reflects RNA integrity it also indexes the quality of the reactions from total RNA to cRNA applied to the chips. However, it is clear that yield and RNA integrity have different relationships with the post-chip QC factors. In our experiment where all reactions were carried out by the same person in large batches it is unlikely that there were large variations in yield due to technical factors. Our study is limited by having no systematic technical replicates, for as in most such studies this would have been too expensive. The only RNA sample that was re-hybridised to an A chip, having been re-generated from RNA, was rated as poor and failed on both occasions.

It is useful to distinguish between the various facets of the catch-all term 'quality'. In chronological order: there is first the condition of the starting RNA; next is the calibre of the experimental process and resulting hybridisation; finally comes the acceptability of the resulting expression measures, including identification of outliers.

We can think of the first four principal components as providing a grouping of post-chip measures, with each component representing a different aspect of quality (see Table 5): the first component reflects variables gauging *array outliers*, the second comprises variables assessing *array adjustment*, the third contains variables measuring *hybridisation noise*, and the fourth consists of the set of variables related to *RNA integrity*. Interestingly, these components correspond roughly to the three aspects mentioned above, but in the reverse order. The first and second components together give insight into the outlier status of a chip when it is considered as part of a set of chips. The component explaining most variability contains variables providing numerical assessments for outlier identification. A related but somewhat distinct aspect is given by an assessment of how far off the chip is from the others, or how the signal would need to be adjusted to make it more like the rest of the chips in the set; this is provided by variables strongly represented in the second component. The third and fourth components, respectively, reflect directly the second (hybridisation) and first (RNA integrity) areas of quality.

It is notable that none of the quality assessment procedures have measures in all four categories covered by the principal components. The different algorithms appear to mostly view quality from different, though overlapping, perspectives. The measures provided by MAS 5.0 are most prominent in the noise and integrity aspects, but also touch on array adjustment. The RMA-QC measures dominate in outlier identification, but also include array adjustment; integrity is tangentially included in the affy package [21] through the slope and corresponding p-value. DChip measures also focus on outlier identification and array adjustment, but include a noise variable as well [9].

These different softwares are often used in conjunction with one another, as indeed we did in our original analysis. Presumably it is hoped that this increases the chance of picking out all of the important variation, although it may also increase the chances of excluding from the analysis chips with data of sufficient quality.

The relationships revealed by the canonical correlations confirm that RNA perceived by subjective assessment or RIN to be of high quality correlates strongly with post-chip measures of 3'/5' integrity in the first canonical correlation. This is reflected in the ability of B_ACTIN35 and GAPDH35 to predict RIN retrospectively. RMA and dChip measures do not explicitly index the 3'/5' ratios, seen in the relationship between RIN and the affy package RNADEG_SL, that the canonical correlations reveal to be highly correlated: we did not use the RNADEG_SL in our original decisions about which chips to exclude from analysis. In retrospect it only identified 1 out of the 6 samples that we subsequently decided to drop from the analysis in our original method of exclusion and in fact identified two other chips that we decided on balance to include.

However, the scaling factor and median log ratios of RMA expression are also strongly related to RNA integrity variables, indicating that scaling factors or bias should be as consistent as possible across all chips in an experiment. The relationship of SF to RIN is evidenced by its ability to predict RIN without invoking the measures of 3'/5' ratios. This is entirely logical as higher RNA integrity is related to higher yield of cRNA and to a signal better able to be distinguished from the noise or background. Thus scaling factors will be smaller and percentage present calls higher if the RNA is of good quality, as shown by the first canonical correlation. The less adjustment that needs to be applied between different chips, the more likely that a clearly interpretable signal will be obtained from an experiment. That is, there will be increased sensitivity to detect changes. Including data from a poor quality chip affects the numbers of probesets considered to be differentially expressed very markedly; this effect is amplified with smaller numbers of biological replicates. An FDR adjustment [22] can control the false discovery levels in the presence of poor quality chip data, but chips of poor quality appear to substantially increase the number of genes called as false negatives, as does reducing the numbers of biological replicates [23]. This is a result of increased variance reducing the power to detect differential expression [24,25]. Archer et al. [26] suggest that one way of rescuing some data from these poor quality chips is to consider only those probesets for which all probes fall within a defined distance of the 3' end of the gene, although this fails to take into account the complexity of RNA degradation, which is influenced by mRNA higher order structure as well as length [27,28].

All the other variables in the first canonical correlation have correlations of < 0.4 but users should note that lower RNA integrity and yield predict lower %P calls, lower median intensity and higher measures of signal variation. The second canonical correlation shows much weaker relationships and a big difference between RIN and SUBQUAL relationships to the post-chip variables. Good yields combined with poor subjective quality appear to predict higher background measures as though the entire signal, including the noise, has been amplified.

## Conclusion

Our results indicate that, as expected, close attention should be paid to the RNA integrity of the original sample, as indexed subjectively or using RIN. In addition, if cRNA yields are not consistent, despite consistent RNA quality, then it may well be worth regenerating the sample from total RNA before running it on a chip in order to improve the yield. Yield which does not subsequently improve may indicate that the sample should be abandoned as it will not produce sound results. PMI itself appears not to be important in determining the quality of final GeneChip data, but it is weakly related to RNA quality and may index some of this variation. Thus it may only be necessary to exclude samples before hybridisation if their RNA quality is insufficient. As determining RNA quality is the first step in running such an experiment this is clearly useful to know. Of the post-chip measures, RNADEG_SL appears to index RNA integrity better than the 3'/5' measures but both reflect the integrity of the original RNA sample that is known prior to the hybridisation step. Post-chip measures of the RNA integrity aspect of quality are most useful for indicating when there has been poor control of the RNA quality subsequent to its initial integrity assessment, or for reinterpreting publicly available data to enable prediction of its likely quality. In practice, for this experiment, expression data from chips that passed our initial post-chip QC measures were derived from samples with a RIN > 5.5.

It would be useful to identify a relationship between RNA quality, cRNA yield and the subsequent quality of expression data in order to make informed decisions about RNA samples to include in a microarray expression study. However, in this study we have found that the post-chip variables having the strongest association with quantities measurable before hybridisation are those reflecting RNA integrity. Other aspects of quality, such as noise measures (reflecting the execution of the assay) or measures reflecting data quality (outlier status and array adjustment variables) are not well predicted by the variables we were able to determine ahead of time. To the extent that random variation affects chip hybridisation, this finding is not very surprising. However, we do not rule out the possibility that there are other variables measurable pre-hybridisation which may be better associated with expression data quality measures. Uncovering such connections could create savings on costly microarray experiments by eliminating poor samples before hybridisation. We therefore encourage investigators to keep careful track of potentially relevant variables so that further studies may continue to shed light on features predictive of array quality.

## Methods

### Expression dataset

A large set of post mortem brain samples (N = 134) that had previously been included in an analysis of gene expression using Human Genome U133A arrays (Affymetrix) were included in the current study (Table 9) [6]. These samples, representing three different brain regions from male and female cases (N = 44) aged 34 to 94, were prepared and processed according to standard protocols (GeneChip^® ^Expression Analysis Protocol, Rev. 2, March 2003, Affymetrix). Briefly, total RNA was extracted using TRIzol (Invitrogen) followed by RNeasy column cleanup (Qiagen) using the manufacturers' protocols. 10 μg total RNA from each sample was used to prepare biotinylated fragmented cRNA, with products from Affymetrix. Arrays were hybridized for 16 h in a 45°C incubator with constant rotation at 60 rpm. Chips were washed and stained on the Fluidics Station 400, and scanned using the GeneArray^® ^2500, according to the GeneChip^® ^Expression Analysis Protocol, Rev. 2, March 2003 (Affymetrix). All RNA extractions and reactions were prepared using master mixes and batches of 8 and 24 respectively. Arrays were processed in batches of 16. All reactions and array hybridisations were carried out by the same person. The U133A GeneChips came from two manufacturing batches.

Gene expression was quantified by robust multi-array analysis (RMA) [11] using the affy package [12], available as part of the Bioconductor project [10]. Quality measures were obtained from MAS 5.0 rpt files [8], dChip software [9], Bioconductor package affyPLM [21] and specialised routines for obtaining QC measures from robust regression [29]. The data are available as part of GEO accession GSE3790.

### Description of the variables assessed

A number of pre- and post-array variables were selected for analysis on the basis of their predicted contribution to quality control assessment at various points in the procedure, from sample processing to expression data (Table 1 and below).

### Pre-chip variables

#### Post mortem interval

(PMI) Post mortem interval was the time from death to tissue preservation, in hours.

#### Subjective RNA quality

(SUBQUAL) 300 ng of total RNA was run on a 2100 Bioanalyzer (Agilent Technologies). A pre-defined four category subjective rating of RNA quality for all samples was made independently by an experienced biologist (AKH) and checked by another (RLC): in the few cases of disagreement (<5% of the total) the sample trace was re-evaluated and a consensus was reached. In performing this assessment, attention to the following features of the total RNA trace were made: ribosomal peak definition, baseline flatness, and whether there were increased low molecular weight species. Traces were used to classify RNA quality as excellent, good, fair or poor. For subsequent statistical analysis a value of 4 was assigned to samples considered excellent, 3 to good, 2 to fair and 1 to poor total RNA integrity. This rating was used to select samples for further processing. Since a limited number of chips were available for the experiment, a minority of samples rated as 1 were not processed further, (although not all samples rated as 1 were excluded at this point) (Table 9 and Figure 1).

#### Objective RNA quality RIN

(RIN) Subsequent to the completion of the experiment, 2100 Bioanalyzer Expert software became available that enabled automatic assessment of RNA quality for mammalian eukaryotic total RNA (Agilent Technologies). The RNA integrity number (RIN) assesses RNA integrity on a scale from 0 (low integrity RNA) to 10 (high integrity RNA). The algorithm for generating a RIN number for a given RNA sample is based on the entire electrophoretic trace of the RNA sample. It uses an artificial neural network based on the determination of the most informative features that can be extracted from the traces out of 100 features identified through signal analysis. The selected features which collectively capture the most information about the integrity levels include the total RNA ratio (ratio of area of ribosomal bands to total area of the electropherogram), the height of the 18S peak, the fast area ratio (ratio of a defined area in the low molecular weight range to the total area of the electropherogram) and the height of the lower marker [7]. RINs were generated for all samples retrospectively with 210 Bioanalyzer software (release B.02.02.SI238) using 2100 Bioanalyzer traces made at the time of the experiment. The algorithm within the Agilent software has been trained to identify electropherogram traces that have an unusual shape and will not return a RIN value in such cases [7]. A RIN number for 2 samples could not be determined and these were therefore excluded from the current analysis.

#### cRNA yield

(YIELD) A standard amount of total RNA (10 μg) was used to carry out cDNA and subsequent cRNA reactions for each sample. The yield of adjusted cRNA (corrected for total RNA input) was used as a pre-chip parameter and reflects both RNA quality and the technical variation of the sample preparation.

### Post-chip variables

#### (Affymetrix) quality control measures [8]

##### Background

(BG) Background is a measure of the fluorescent signal on the array due to non-specific binding and autofluorescence from the array surface and the scanning wavelength (570 nm). A high background may indicate the presence of impurities such as cell debris or salts. This non-specific binding causes a low signal to noise ratio, resulting in reduced sensitivity for the detection of low expressing mRNA.

##### Noise (Raw Q)

(RAWQ) Raw Q assesses the pixel to pixel variation within each probe cell. Electrical noise from the scanner and sample quality can both contribute to Raw Q.

##### Noise

(NOISE) Noise is calculated by dividing the array into 16 zones. The standard deviation of the lowest 2% of signal is calculated for each zone and then the average value for all zones is determined. Noise is used to calculate background adjusted signal values by taking a weighted average of the zone-specific noise levels.

##### Percentage present

(PC_PRESENT) The number of probesets called "present" relative to the total number of probesets on the array as a percentage. A probeset is determined to be present, marginal or absent by a statistical algorithm within the MAS 5.0 software.

##### Scaling Factor

(SF) When global scaling is performed, the overall intensity for each array is determined and is compared to a Target Intensity value in order to calculate the appropriate scaling factor. The Scaling Factor (calculated by the global method) should be comparable between arrays.

##### 3'/5' ratios of housekeeping genes (β-Actin, GAPDH)

(B_ACTIN35, GAPDH35 respectively) Expression values for probesets specific to the 5', middle, or 3' portion of the *ACTB *and *GAPDH *transcripts are calculated from the chip. The 3' and 5' probesets' expression values are divided to give a ratio of 3'/5' mRNA representation.

#### dChip software quality control measures [9,30]

##### PM/MM difference array outlier algorithm

(DCHIP_AR_OUTLIER) The algorithm identifies outliers as a result of image contamination or saturated PM or MM signals. It cross-references one array with all the other arrays in an experiment using modelling of both perfect match (PM) and mismatch (MM) probe information at the probeset level. It flags an array as an outlier if > 5% of the probesets on that array are outliers relative to all other arrays in the experiment and recommends discarding arrays with >15% outlier probesets from analyses.

##### Single outlier algorithm

(DCHIP_SING_OUTLIER) The algorithm also determines single outliers, individual probes within a probeset. These are most likely due to cross-hybridization to non-target or alternatively spliced genes.

##### %P

(DCHIP_PCCALL) Probesets that the dChip algorithm considers to be greater than zero, i.e. expressed, are determined as a percentage of the total number of probesets, similar to the Affymetrix %P.

##### Median intensity

(MEDINT) The median intensity is determined across the array.

#### Bioconductor affy and affyPLM package quality control measures [13]

From affyPLM [21]

##### Median NUSE

(MED_NUSE) Standard error (SE) estimates for each probeset on the array are taken and adjusted so that the median standard error across all arrays is equal to 1. Arrays with elevated SE relative to other arrays are typically of lower quality. Boxplots of these values are used to compare arrays.

##### Relative Log Expression (RLE)

Values are computed for each probeset by comparing the expression value on each array against the median expression value for that probeset across all arrays. The assumption is that most genes have constant expression across arrays, and thus have RLE values close to zero. Deviations from this are assessed using boxplots. A number of statistics within this analysis can be assessed as variables including: inter-quartile range of log ratios (IQR_LR), median (or bias) log ratio (B_LR), interquartile range plus absolute median of log ratios (IQRplusAbsB_LR) andthe coefficient of variation of log ratios (CV_LR) whichsummarise distributions of log ratios, or relative log expression, at the chip level. The relative log expression for each probe set is obtained by subtracting a baseline log expression from the log expression of each probe set. Due to computing limitations, we separated our experiment into two sets of chips. We used one series to fit the RMA model, and then applied the fitted model to all chips. Log ratios are computed using two different sets of chips for baseline. In LR1 the baseline is the probe set median log expression for the fitting set of chips. In LR2, the baseline is the median log expression from the group under study itself.

##### RNA degradation plot (gradient)

(RNADEG_SLOPE) Within each probeset, probes are numbered directionally from the 5' end to the 3' end. Probe intensities are averaged by probe number, across all probesets. Outlying arrays are identified as those with a different gradient to the majority of plots within the experiment. The *P-*value from the linear regression fit for the RNA degradation plot is also assessed as a variable (PVAL_SLOPE).

### Statistical analysis

All analyses were performed on the combined dataset for all three brain regions (cerebellum, caudate nucleus, frontal cortex) from a subset of samples (N = 112) for which data was available for all variables (U133A arrays). Each of the pre- and post- chip variables were adjusted by "brain region" in order to remove the effect of the latter, using the univariate general linear modelling technique where the "brain region" variable was fitted as a fixed effect. The residuals were considered as continuous variables and used for further analysis. The adjusted variables were assessed for normality using Skewness and Kurtosis measures in SPSS and distributions were considered non-normal if they did not fall between 0–1.

Factor analysis was performed on 22 adjusted post-chip variables (see above) using SAS PROC FACTOR. The analysis is based on the Spearman correlation matrix generated using the SAS PROC CORR. Initial factors were extracted using the principal component method and rotations were then performed by the VARIMAX method. To assess relationships between the "pre-chip" and "post-chip" multidimensional variables (4 and 22 dimensions respectively), canonical correlation analysis was performed using the SAS PROC CANCOR. As above, the analysis was based on Spearman correlation matrix (SAS PROC CORR).

For predicting RIN from post-chip variables, we assume a linear model. High multicollinearity between variables precludes formulation of a robust, interpretable model to predict RIN based on post-chip variables. We thus considered simple models containing one or two predictors.

Robust linear regression modeling [16,17] of RIN based on single variables highly correlated with RIN was used to create simple prediction rules. We also used ordinary least squares linear regression for comparison; results from both methods were quite similar.

## Authors' contributions

Laboratory work was carried out by AKH, and judgment of RNA quality by AKH and RL-C. AKH and GH collated the data. VM and DRG did the main statistical analysis and FC, AA, CK and SBD all contributed to the statistical and bioinformatic analysis of data. Study design was by AKH, JMO, CK, SJA, RLMF, RL-C and LJ. RLMF supplied the tissue and the clinical information about the samples. LJ, DRG and AKH interpreted the data and took the primary role in writing the manuscript. All authors read and commented upon the manuscript.

## Supplementary Material

Additional File 1 Boxplots illustrating the summary statistics for the pre- and postchip variables from Table 1. Click here for file

Additional File 2 Scatterplots of the pre- and post-chip variables for all chips studied showing the behaviour of the identified outlying chips in Table 3. Click here for file

## Figures and Tables

**Table 1:** Pre- and post-chip variables assessed in the current study on U133A arrays, with indications of the recommended acceptable range (where available) and summary statistics determined for samples in the current study. * None of the chips had significant spatial artifacts of hybridisation (e.g. scratches, uneven hybridisation) as assessed by visual inspection of the chip following hybridisation and by assessment of array and single outlier status across the chips. MAD is the median absolute deviation.

Variable	Variable (abbreviated)	Recommended acceptable range	Summary statistics (current experiment)			
			
			Median	MAD	Range	
Pre-chip variables						
Post mortem interval hours	PMI	empirical	12	3	4	29
Subjective RNA quality 4 point scale	SUBQUAL	empirical	3	1	1	4
RNA Integrity Number (RIN) 0 to 10	RIN	empirical	7.8	0.9	4.3	9.5
Adjusted cRNA yield (μg)	YIELD	>15 μg	57	7	30	83

Post-chip variables: assessed within MAS 5.0 (Affymetrix)						

Background	BG	< 100	101.3	11.99	72.32	438.41
Noise (Raw Q)	RAWQ	1.5 – 3.0	3.73	0.35	2.83	9.52
Noise	NOISE	empirical	5.76	0.82	3.85	38.2
%P	PC_PRESENT	25 – 50%	47	2	24	53
Scaling Factor	SF	< 10	2.18	0.385	1.08	7.68
3'/5' ratio of β-Actin	B_ACTIN35	< 3	1.6	0.26	0.98	55.87
3'/5' ratio of GAPDH	GAPDH35	< 1.25	1.2	0.16	0.77	31.62

Post-chip variables: assessed within dChip [9]						

PM/MM difference array outlier algorithm*	DCHIP_AR_OUTLIER	< 5%	0.37	0.2	0.04	47.59
PM/MM difference single outlier algorithm*	DCHIP_SING_OUTLIER	< 5%	0.09	0.03	0.04	2.3
%P	DCHIP_PCCALL	25 – 50%	58.4	1.6	36.2	63.4
Median intensity	MEDINT	empirical	358	43.5	231	1620

Post-chip variables: assessed within Affy, Bioconductor, developed by Collin, 2005 [13]						

Median NUSE	MED_NUSE	< 1.05	1.01	0.01	0.98	1.26
IQR.LR1	IQR_LR1	empirical	0.23	0.03	0.15	1.04
B.LR1	B_LR1	empirical	0.005	0.02	-0.11	0.24
IQRplusAbsB.LR1	IQRplusAbsB_LR1	empirical	0.24	0.04	0.15	1.13
CV.LR1	CV_LR1	empirical	6.41	3.9	0.01	35.91
IQR.LR2	IQR_LR2	empirical	0.18	0.03	0.11	0.98
B.LR2	B_LR2	empirical	0	0.01	-0.1	0.22
IQRplusAbsB.LR2	IQRplusAbsB_LR2	empirical	0.2	0.035	0.12	1.02
CV.LR2	CV_LR2	empirical	3.97	3.225	0	33.8

Post-chip variables: assessed within Affy, Bioconductor [13]						

RNA degradation plot (gradient)	RNADEG_SLOPE	empirical	2.27	0.27	0.86	3.96
RNA degradation plot (P- value from the linear regression)	PVAL_SLOPE	empirical	2.67E-08	2.60e-8	4.21e-12	0.0049

**Table 2:** Correlations between pre-chip variables. Matrix of correlations (over the main diagonal) and p-values for difference from 0 (under the main diagonal) between pre-chip variables.

	YIELD	PMI	RIN	SUBQUAL
YIELD	*	-0.22	0.29	0.34
PM	0.018	*	-0.19	-0.21
RIN	0.002	0.043	*	0.71
SUBQUAL	0.0003	0.025	0.000001	*

**Table 3:** Pre- and post chip variables for samples flagged as potential outliers on at least one post-chip quality control measure. Samples were identified as outliers as judged empirically within the experiment and detailed in the results. Samples (*) were excluded from expression analysis. Samples from cases with prolonged agonal state (†). CB, cerebellum; CN, caudate nucleus; MC, motor cortex.

Pre-chip variables									
Sample	PMI		SUBQUAL		RIN		YIELD		

H104 CB	14		3		7.4		77		
H122 CB*^†^	9		2		8.1		68		
H85 CB*^†^	10		3		7.4		65		
HC79 CB*^†^	4		4		8.1		44		
H123 CN*	7.5		3		7.6		66		
HC61 CN	6		3		7.1		59		
H131 MC*	13		4		9.1		48		
HC71 MC*	5		1		4.3		36		
HC52 MC	23		1		6.5		56		
HC55 MC	20		1		6.6		44		

Post-chip variables: assessed within MAS 5.0 (Affymetrix)									

	BG	RAWQ	NOISE	PC_PRESENT		SF	B_ACTIN35		GAPDH35

H104 CB	271	7.5	13.9	42		2.2	1.7		1.2
H122 CB*^†^	438	9.5	34.8	36		1.9	1.7		1.0
H85 CB*^†^	92	3.6	5.7	49		2.1	1.8		1.1
HC79 CB*^†^	87	3.4	5.5	45		1.7	55.9		31.6
H123 CN*	103	3.7	7.6	30		4.1	2.4		0.8
HC61 CN	119	4.0	5.2	43		4.0	1.5		1.2
H131 MC*	250	8.4	38.2	24		1.1	1.6		1.1
HC71 MC*	107	3.6	4.7	26		7.7	4.0		4.1
HC52 MC	122	4.2	6.0	39		3.6	3.9		2.1
HC55 MC	98	3.4	4.7	41		3.4	2.6		2.0

Post-chip variables: assessed within dChip (Li and Wong, 2001)									

	DCHIP_AR_OUTLIER		DCHIP_SING_OUTLIER		DCHIP_PCCALL		MEDINT		

H104 CB	2.71		0.61		55		597		
H122 CB*^†^	14.72		1.85		41		1199		
H85 CB*^†^	6.92		0.40		61		358		
HC79 CB*^†^	12.75		0.38		55		327		
H123 CN*	30.48		1.72		42		369		
HC61 CN	4.05		0.58		57		287		
H131 MC*	47.59		2.30		36		1620		
HC71 MC*	19.66		1.23		39		266		
HC52 MC	1.70		0.20		52		331		
HC55 MC	3.19		0.29		53		294		

Post-chip variables: assessed within Bioconductor packages affy, affyPLM and specialised code [15, 22]									

	MED_NUSE	IQR_LR1	B_LR1	IQRplusAbsB_LR1	CV_LR1	IQR_LR2	B_LR2	IQRplusAbsB_LR2	CV_LR2

H104 CB	1.06	0.27	-0.048	0.32	17.3	0.27	-0.029	0.30	10.7
H122 CB*^†^	1.24	0.66	-0.105	0.76	16.0	0.64	-0.098	0.74	15.2
H85 CB*^†^	1.08	0.42	0.008	0.43	1.9	0.38	0.014	0.40	3.7
HC79 CB*^†^	1.12	0.56	-0.001	0.56	0.2	0.56	0.000	0.56	0.1
H123 CN*	1.26	0.67	0.241	0.91	35.9	0.65	0.220	0.87	33.8
HC61 CN	1.06	0.30	0.025	0.33	8.4	0.25	0.000	0.25	0.0
H131 MC*	1.26	1.04	-0.050	1.09	4.8	0.98	-0.040	1.02	4.1
HC71 MC*	1.2	0.96	0.165	1.13	17.2	0.87	0.150	1.02	17.3
HC52 MC	1.06	0.43	0.063	0.49	14.5	0.34	0.039	0.38	11.3
HC55 MC	1.07	0.42	0.039	0.46	9.4	0.34	0.015	0.35	4.4

Post-chip variables: assessed within affy, Bioconductor									

	RNADEG_SLOPE					PVAL_SLOPE			

H104 CB	2.40					3.8 × 10^-9^			
H122 CB*^†^	0.86					4.9 × 10^-3^			
H85 CB*^†^	2.22					1.2 × 10^-7^			
HC79 CB*^†^	3.96					1.0 × 10^-9^			
H123 CN*	1.61					3.2 × 10^-7^			
HC61 CN	2.40					9.7 × 10^-9^			
H131 MC*	1.17					2.2 × 10^-5^			
HC71 MC*	2.67					6.0 × 10^-10^			
HC52 MC	3.61					4.2 × 10^-12^			
HC55 MC	3.20					3.2 × 10^-11^			

**Table 4:** Four main principal components explain ~75% of the variance in post-GeneChip QC measures)

Component	% variance explained	Cumulative variance explained
1	31.6	31.6
2	17.8	49.4
3	13.4	62.8
4	12.1	74.9

**Table 5:** Rotated component matrix for post-chip variables (U133A arrays, all samples)

QC measure	Component			
	
	1	2	3	4
IQR_LR1	0.934			
IQR_LR2	0.928			
IQRplAbsB_LR1	0.936			
IQRplAbsB_LR2	0.938			
DCHIP_AR_OUTLIER	0.771			
MED_NUSE	0.769			
DCHIP_SING_OUTLIER	0.557			
CV_LR1	0.418			
CV_LR2	0.327			
B_LR1		0.905		
B_LR2		0.855		
SF		0.734		
DCHIP_PCCALL		-0.848		
PC_PRESENT		-0.866		
NOISE			0.959	
RAWQ			0.934	
BG			0.928	
MEDINT			0.874	
B_ACTIN35				0.960
GAPDH35				0.946
PVAL_SLOPE				0.789
RNADEG_SL				0.427

Values less than 0.4 are not shown unless they are the largest for the corresponding variable

**Table 6:** Canonical correlation analysis to explore the relationships between two sets: a set of 4 pre-chip and a set of 22 post-chip variables

Canonical Correlation		Approximate Standard Error	Significance
1	0.838	0.028	< 0.0001
2	0.593	0.061	0.012
3	0.500	0.072	0.154
4	0.454	0.075	0.263

**Table 7:** Canonical factor loadings of the pre-chip variables corresponding to the first and the second canonical correlations

Pre-chip QC	Canonical variables	
	
	Pre-chip1	Pre-chip2
YIELD	-0.526	0.363
PMI	0.140	0.112
RIN	-0.934	0.099
SUBQUAL	-0.847	-0.521

**Table 8:** Canonical factor loadings of the post-chip variables corresponding to the first and the second canonical correlations

Post-chip QC	Canonical variables	
	
	Post-chip1	Post-chip2
RNADEG_SL	0.845	
B_ACTIN35	0.532	0.280
GAPDH35	0.513	0.280
SF	0.497	
B_LR1	0.364	
B_LR2	0.345	
IQR_LR1	0.289	
IQR_LR2	0.366	
IQRplAbsB_LR1	0.288	
IQRplAbsB_LR2	0.343	
MED_NUSE	0.337	
DCHIP_AR_OUTL	0.337	
DCHIP_SING_OUT	0.313	
DCHIP_PCCALL	-0.381	
MEDINT	-0.377	
PC_PRESENT	-0.339	
CV_LR1		
CV_LR2		0.376
BG		0.336
NOISE	-0.284	0.275
RAWQ	-0.287	0.303
PVAL_SLOPE		0.538

Values less than 0.25 are not shown

**Table 9:** The number of samples included at various steps in the process from total RNA to analysis of expression and in the current analysis of quality control measures

**Sample numbers**	**Cerebellum**	**Caudate nucleus**	**Motor Cortex**	**Total**
**RNA**	45	45	44	134
**run on chips**	39	41	37	117
**included in array analysis [5]**	36	41	35	112
**included in QC correlations**	36	38	35	117
