# Supplementary material for: Assessment of the relationship between pre-chip and post-chip quality measures for Affymetrix GeneChip expression data
Source: BMC Bioinformatics. 2006 Apr 19;7:211. doi: 10.1186/1471-2105-7-211 (PMC1524996; doi:10.1186/1471-2105-7-211)
Supplement: Additional File 1 — Boxplots illustrating the summary statistics for the pre- and postchip variables from Table 1. [file 1471-2105-7-211-S1.pdf]

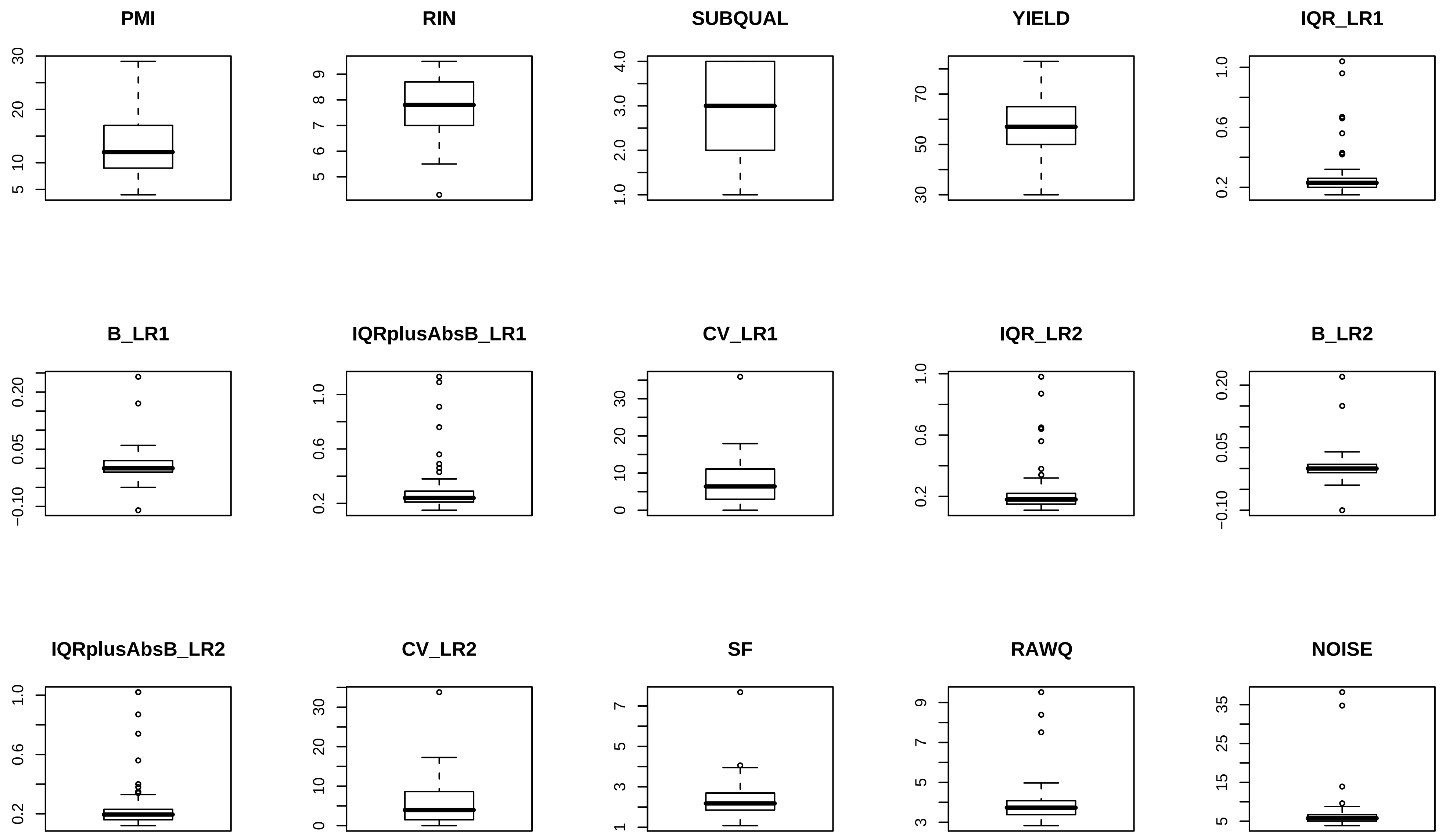

PMI
RIN
SUBQUAL
YIELD
IQR_LR1
B_LR1
IQRplusAbsB_LR1
CV_LR1
IQR_LR2
B_LR2
IQRplusAbsB_LR2
CV_LR2
SF
RAWQ
NOISE

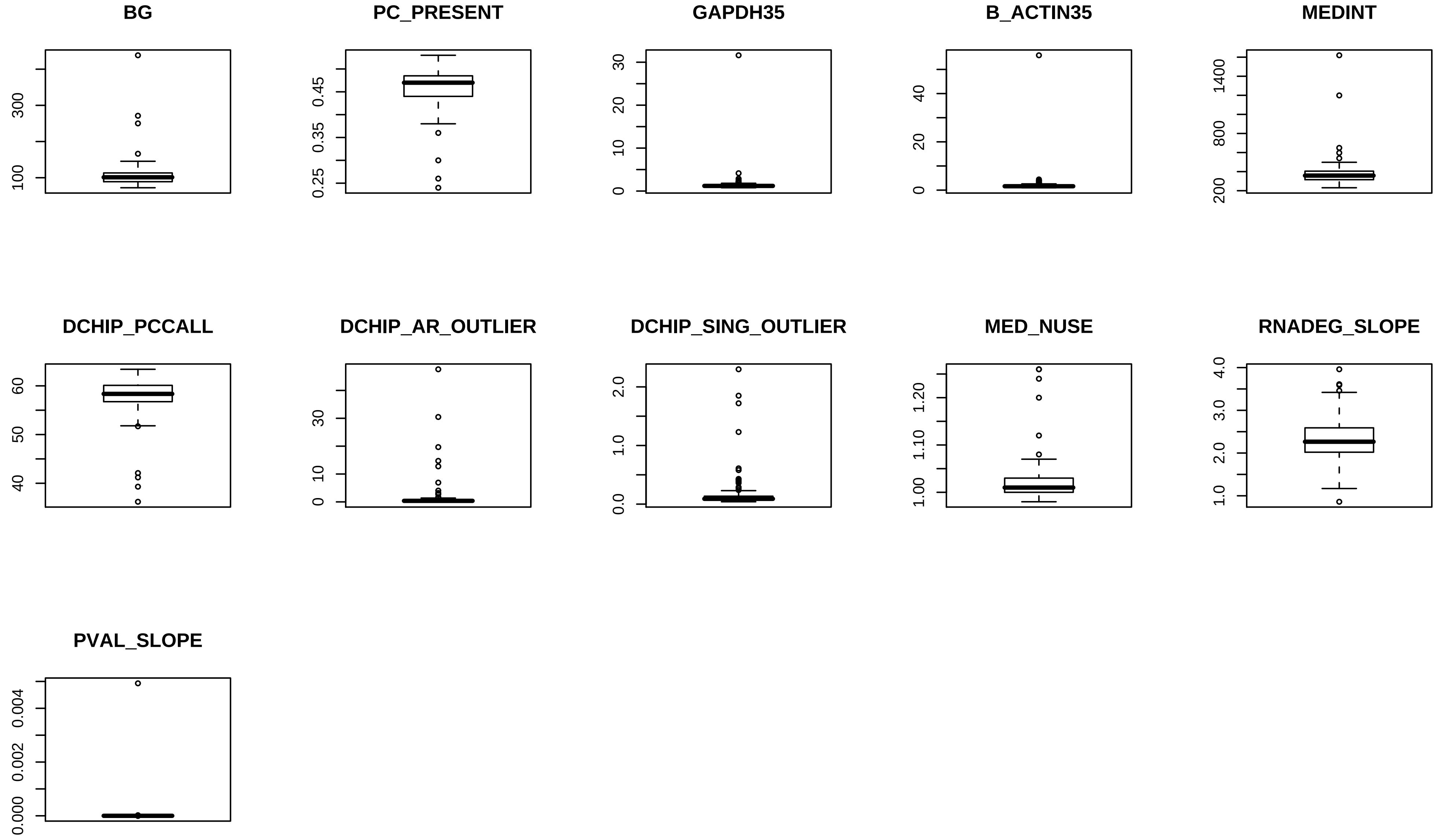
BG
100
300
PC_PRESENT
0.25
0.35
0.45
GAPDH35
0
10
20
30
B_ACTIN35
0
20
40
MEDINT
200
800
1400
DCHIP_PCCALL
40
50
60
DCHIP_AR_OUTLIER
0
10
30
DCHIP_SING_OUTLIER
0.0
1.0
2.0
MED_NUSE
1.00
1.10
1.20
RNADEG_SLOPE
1.0
2.0
3.0
4.0
PVAL_SLOPE
0.000
0.002
0.004
