# Supplementary figures and images for: Assessment of the relationship between pre-chip and post-chip quality measures for Affymetrix GeneChip expression data
Source: BMC Bioinformatics. 2006 Apr 19;7:211. doi: 10.1186/1471-2105-7-211 (PMC1524996; doi:10.1186/1471-2105-7-211)

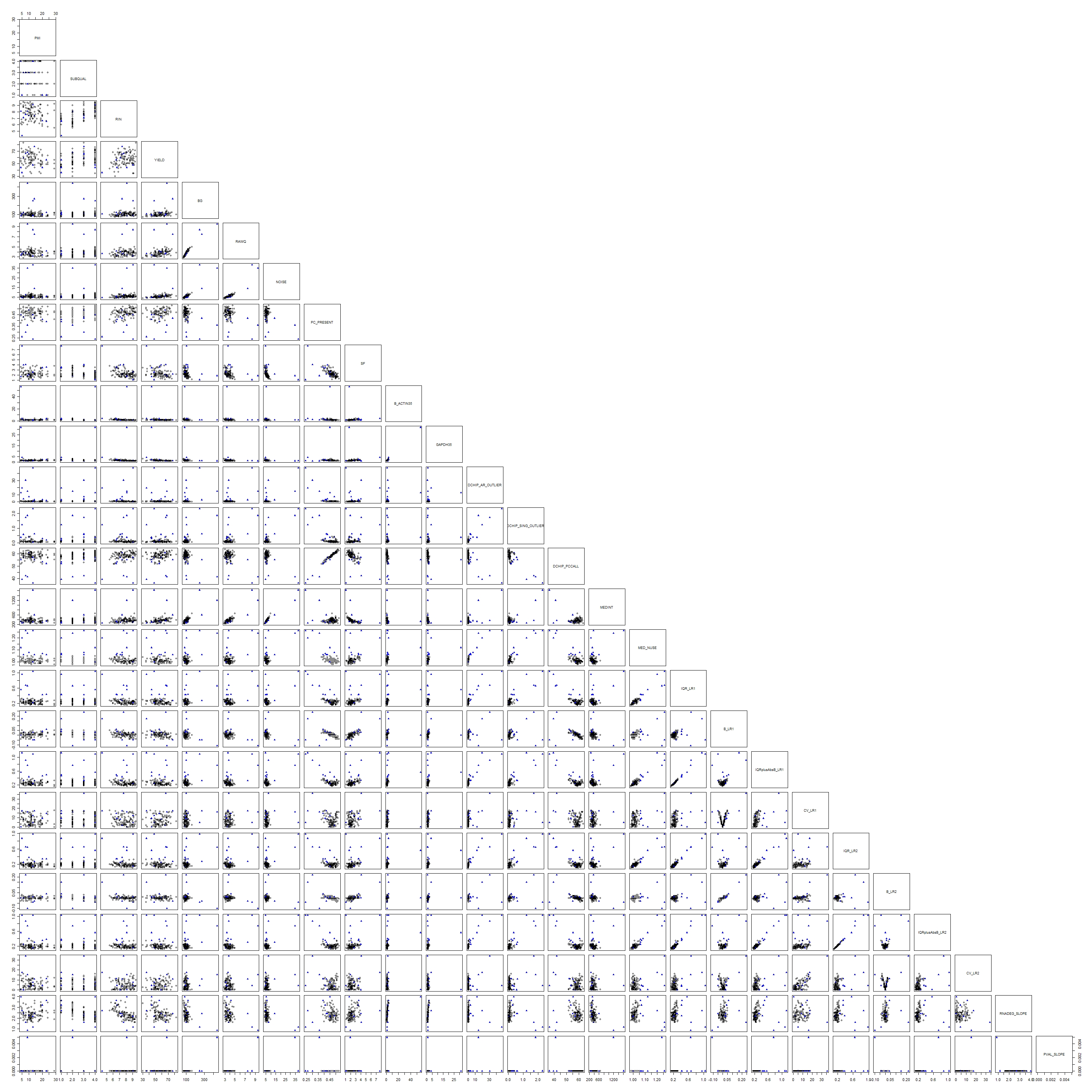

Supplement: Additional File 2 — Scatterplots of the pre- and post-chip variables for all chips studied showing the behaviour of the identified outlying chips in Table 3. [file 1471-2105-7-211-S2.jpeg]
